# Initial activation of EpCAM cleavage *via *cell-to-cell contact

**DOI:** 10.1186/1471-2407-9-402

**Published:** 2009-11-19

**Authors:** Sabine Denzel, Dorothea Maetzel, Brigitte Mack, Carola Eggert, Gabriele Bärr, Olivier Gires

**Affiliations:** 1 Clinical Cooperation Group Molecular Oncology, Helmholtz-Zentrum München, German Research Center for Environmental Health, and Head and Neck Research Dept. Ludwig-Maximilians-University of Munich, Germany; 2 Department of Otorhinolaryngology, Head and Neck Surgery, Großhadern Medical Center, Ludwig-Maximilians-University of Munich, Marchioninistr. 15, 81377 Munich, Germany

## Abstract

**Background:**

Epithelial cell adhesion molecule EpCAM is a transmembrane glycoprotein, which is frequently over-expressed in simple epithelia, progenitors, embryonic and tissue stem cells, carcinoma and cancer-initiating cells. Besides functioning as a homophilic adhesion protein, EpCAM is an oncogenic receptor that requires regulated intramembrane proteolysis for activation of its signal transduction capacity. Upon cleavage, the extracellular domain EpEX is released as a soluble ligand while the intracellular domain EpICD translocates into the cytoplasm and eventually into the nucleus in combination with four-and-a-half LIM domains protein 2 (FHL2) and β-catenin, and drives cell proliferation.

**Methods:**

EpCAM cleavage, induction of the target genes, and transmission of proliferation signals were investigated under varying density conditions using confocal laser scanning microscopy, immunoblotting, cell counting, and conditional cell systems.

**Results:**

EpCAM cleavage, induction of the target genes, and transmission of proliferation signals were dependent on adequate cell-to-cell contact. If cell-to-cell contact was prohibited EpCAM did not provide growth advantages. If cells were allowed to undergo contact to each other, EpCAM transmitted proliferation signals based on signal transduction-related cleavage processes. Accordingly, the pre-cleaved version EpICD was not dependent on cell-to-cell contact in order to induce *c-myc *and cell proliferation, but necessitated nuclear translocation. For the case of contact-inhibited cells, although cleavage of EpCAM occurred, nuclear translocation of EpICD was reduced, as were EpCAM effects.

**Conclusion:**

Activation of EpCAM's cleavage and oncogenic capacity is dependent on cellular interaction (juxtacrine) to provide for initial signals of regulated intramembrane proteolysis, which then support signalling via soluble EpEX (paracrine).

## Background

Epithelial cell adhesion molecule EpCAM is a membrane-bound glycoprotein involved in signalling that promotes gene transcription and cell proliferation [1-3]. The high-level over-expression of EpCAM in a plethora of carcinomas [4] led to the use of it as a marker with prognostic quality and as a target for therapeutic strategies [5-7]. Most-recent findings revealed the necessity for regulated intramembrane proteolysis (RIP) for the induction of EpCAM-related signal transduction, which initiates at the plasma membrane [8,9]. EpCAM becomes proteolytically activated *via *cleavage by TACE (tumour necrosis-factor α converting enzyme) and a gamma-secretase complex comprising presenilin 2 (PS2) [8]. After RIP, the intracellular domain of EpCAM (EpICD) is released in the cytoplasm and shuttles into the cell nucleus in a complex with the scaffold protein FHL2 (four and a half lim domain protein 2) and β-catenin. Thereupon, EpICD contacts members of the TCF/Lef family of transcription factors, binds DNA at Lef consensus sites, and induces transcription of target genes, including *c-myc*, cyclins, and genes related to proliferation [2,3,8]. Expression of EpCAM in murine and human embryonic stem (ES) cells revealed essential to the maintenance of the pluripotent and proliferative phenotype *in vitro*. SiRNA-mediated inhibition of mEpCAM expression in ES cells in the presence of factors necessary for a de-differentiated phenotype induced differentiation, reduced proliferation, and diminished expression levels of classical ES cell markers such as Oct3/4 and c-Myc [10,11]. Owing to its mode of action and capacities, EpCAM was termed a "*surface-to-nucleus missile*" [9] that is involved cancer and stem cells' signalling [12].

Both, full-length EpCAM but also EpICD, which is composed of twenty-six amino acids only, rendered HEK293 cells tumourigenic *in vivo *and yielded large tumours with high efficiency after xenotransplantation in SCID mice. Likewise, EpICD alone sufficed to substitute for the deficiency to express EpCAM *in vitro *and supported proliferative signals in the absence of the remaining domains of EpCAM [8]. It is further important to note that the over-expression of EpCAM is part of the signature of cancer-initiating cells at least in human colon, breast, and pancreas carcinomas [13-15]. Thus, the aptitude of EpCAM to regulate gene transcription alongside with the *Wnt *pathway and its strong oncogenic potential pinpoint an important role in cancer, eventually related to the origin of malignancies, *i.e*. cancer-initiating cells.

It is however still not entirely understood how EpCAM cleavage and the subsequent signalling cascades are triggered. First indication for a potential mechanism came from stainings of cell agglomerates, where EpCAM was essentially cleaved at areas of cell-to cell contact [8]. Additionally, it was demonstrated that ectodomain shedding resulted in the formation of soluble EpEX, which is instrumental as a ligand in EpCAM signalling. Treatment of EpCAM-positive cells with a recombinant version of EpEX (rEpEX) induced EpCAM cleavage, suggesting that after an initial releasing trigger (*i.e*. in a juxtacrine fashion), soluble EpEX might provide cells with a paracrine signal, as was shown for L1, EGF-R, TNF-R, and others [16-19].

We assessed the dependency of EpCAM cleavage, signalling, and proliferation for cell-to-cell contacts. EpCAM cleavage and subsequent proliferative signals were observed only in cells grown at sufficient initial density to allow for cell-to-cell contact at the onset of the experiment. Oppositely, cells expressing the cleaved intracellular domain EpICD instead of full-length EpCAM were independent of contacts to neighbouring cells for proper proliferation. Thus, cell-to-cell contact is one initial trigger for RIP of EpCAM and nuclear translocation of the released signalling moiety is mandatory for the induction of gene transcription, and for cellular proliferation.

## Methods

### Antibodies, cell lines, and plasmids

α-EpEX antibody HO.3 [20], α-EpICD antibody (guinea pig antibody raised against the intracellular domain; PSL, Peptide Specialty Laboratories, Heidelberg, Germany), HA-tag (Roche, Heidelberg, Germany), c-Myc, eFABP, Cyclin A and E, ERα (F-10) (Santa Cruz, Santa Cruz, USA) were used. For laser-scanning fluorescence microscopy, dye-coupled Alexa antibodies (Alexa- 488, 594, and 647; Molecular Probes, Karlsruhe, Germany) were used as secondary antibodies.

EpCAM, EpICD, and EpICD-HA cDNAs were cloned into the eukaryotic expression vector pCAG-141 to achieve constitutive expression. Additionally, EpICD was fused to a mutated ligand-binding domain of the human estrogen receptor (ER^T^, kind gift of Prof. Dr. Georg Bornkamm) and cloned into pCAG141. All constructs were expressed in human embryonic kidney cells (HEK293). Induction of nuclear translocation of EpICD-ER^T ^was accomplished with 100 nM 4-hydroxytamoxifen (Sigma, Munich, Germany). Stable cell clones were generated by transfection using MATra (IBA, Göttingen, Germany) and selection with 1 μg/ml puromycin (Sigma, Munich, Germany). HEK293 transfectants, HCT-8 and MCF-7 wild type cells were cultured in DMEM with 10% fetal calf serum.

### Cell counting and doubling time

HEK293 transfectants were plated in 10 cm dishes at different densities (3 × 10^5 ^or 3 × 10^6 ^cells/dish). Cell numbers were assessed at different time points upon trypan blue exclusion assay as indicated. Colon carcinoma cells (HCT-8) and breast carcinoma cells (MCF-7) were plated at different densities as follows: D1: 0,5 × 10^5 ^cells/well in 6-well plates which represented 0.05 × 10^5 ^cells/cm^2^; D2: 3 × 10^5 ^cells/well representing 0.31 × 10^5^ cells/cm^2^; D3: 20 × 10^5 ^cells/well representing 2.18 × 10^5^ cells/cm^2 ^and treated similarly. Doubling times were calculated as described [2]. In order to achieve different cell densities with fix cell numbers, 4 × 10^5 ^and 2 × 10^6 ^cells were plated in culture dishes with increasing areas. D1_4 × 105 _= 0.07 × 10^5^ cells/cm^2 ^and D1_2 × 106 _= 0.14 × 10^5^ cells/cm^2^; D2_4 × 105 _= 0.42 × 10^5^cells/cm^2 ^and D2_2 × 106 _= 0.35 × 10^5 ^cells/cm^2^; D3_4 × 105 _= 2 × 10^5^ cells/cm^2 ^and D3_2 × 106 _= 2.08 × 10^5^ cells/cm^2^.

### Immunoblot and immunoprecipitation

For immunoblot analysis, all cell lines were seeded as described for cell counting. Cells were lysed at the indicated time points in 50 μl lysis buffer (1% Triton X100 in TBS). Amounts of proteins were assessed with the BCA™ Protein Assay Kit (Pierce, Thermo Scientific, Rockford, IL, USA). 50 μg of protein lysate were mixed with SDS-PAGE loading buffer (25 mM TrisHCl pH7, 5% glycerin, 1% SDS, 2% beta-mercaptoethanol, bromphenol blue). Proteins were separated by SDS-PAGE, transferred onto PVDF membranes (Millipore, Bedford, US), and detected using specific antibodies in combination with horseradish peroxidase (HRP)-conjugated secondary antibodies and the enhanced chemiluminescence (ECL) reagent (Amersham Biosciences, Freiburg, Germany).

For immunoprecipitation, cells were lysed in PBS/1% triton X100 and protease inhibitors (Roche, Mannheim, Germany). Precleared cell-free supernatants (100,000 g, 30 min) were incubated at 4°C overnight with protein G beads (30 μl, Amersham Biosciences, Freiburg, Germany) loaded with 1 μg of the EpEX-specific antibody HO.3 [20]. Protein G beads were collected by centrifugation, and the pellets were washed five times in cold lysis buffer. Immunoprecipitates were eluted in SDS-PAGE loading buffer (25 mM TrisHCl pH7, 5% glycerin, 1% SDS, 2% beta-mercaptoethanol, bromphenol blue). Immunoprecipitates were analysed by immunoblotting with EpEX-specific antibodies.

### Laser scanning fluorescence microscopy

HCT-8, and MCF-7 cells and HEK293 transfectants were analyzed with a fluorescence laser scanning system (TCS-SP2 scanning system and DM IRB inverted microscope, Leica, Solms, Germany). If stapled sections were recorded, depth of section was 100-180 nm in average. For EpEX and EpICD detection, cells were fixed according to Brock *et al*. [21] and stained with specific antibodies, followed by Hoechst 33342 labelling of nuclear DNA (Sigma, Munich, Germany). Profiling of EpEX, EpICD, and nuclear DNA localisation was conducted with the Leica LCS Lite software with a minimum of 900 measurement points per cell. Where indicated, HCT-8 cells were treated with with 1 μg rEpEX (recombinantly expressed in yeast, Dr. H. Lindhofer, Trion Pharma, Munich Germany) before confocal microscopy was performed.

## Results

### EpCAM cleavage depends on cell-to-cell contact

**Figure 1:**
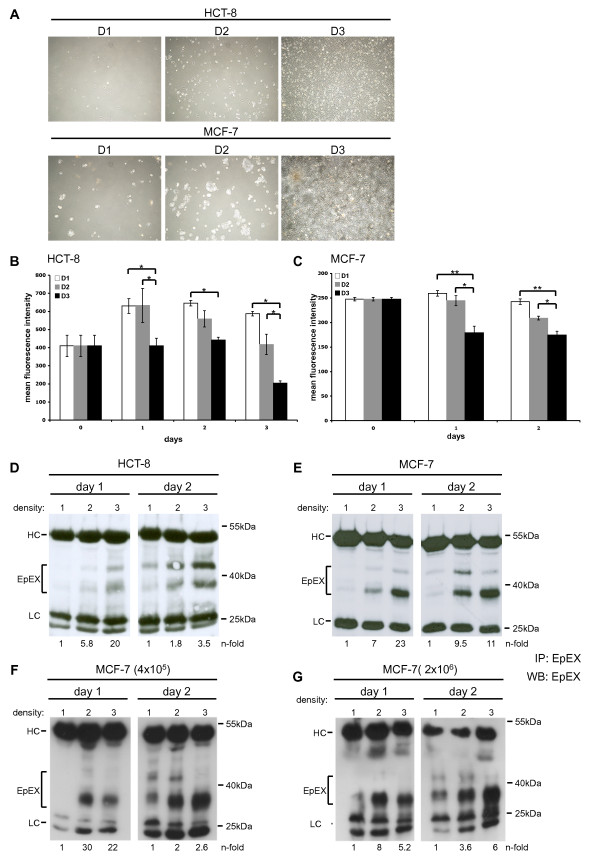
**EpCAM cleavage is dependent on cell-to-cell contact**. (**A**) HCT8 and MCF7 cells were seeded at three different confluencies (D1-3), which resulted in single cells (D1), 20% (D2), and 80% confluency (D3). (**B, C**) Cell surface expression of EpEX was assessed in samples of D1-D3 conditions over a time period of 2-3 days. Shown are the mean fluorescence intensity ratios of EpEX/control with standard deviations from three independent experiments for HCT8 (**B**) and MCF7 cells (**C**). Significant differences are marked (* p < 0.05; ** p < 0.005). (**D, E**) Soluble EpEX was assessed in supernatants of HCT8 (**D**) and MCF7 (**E**) cells in samples of D1-3 conditions. Equal amounts of protein from cell-free supernatants were immunoprecipitated (IP) with EpEX-specific antibodies and EpEX detected by immunoblotting with specific antibodies in combination with HRP-conjugated secondary antibody (marked EpEX). (**F**) MCF7 cells were plated at 4 × 10^5 ^(left panels) or 2 × 10^6 ^(right panels) on increasing areas corresponding to D1, 2, and 3. At day 1 and 2, cell-free supernatants were processed as in E. Levels of EpEX were assessed by image densitometry, normalised for the amount of heavy chain, and values for D1 set to one for a reference. EpEX levels are given n-fold of D1. Shown are representative results from three independent experiments. HC: heavy chains of IP antibodies. LC: light chains of IP antibodies.

HCT8 (colon) and MCF7 (breast) carcinoma cells were seeded at 0.5 × 10^5 ^(D1 = 0,05 × 10^5^/cm^2^), 3 × 10^5 ^(D2 = 0,31 × 10^5^/cm^2^), and 20 × 10^5 ^(D3 = 2,18 × 10^5^/cm^2^) cells in a six well format. Under those conditions, initial cell densities represented single cells (D1), approximately 30% confluency (D2), and up to 90% confluency (D3) for each cell line (Figure 1A). Cell surface expression of EpCAM was monitored for each cell density over a time period of two days by flow cytometry with antibodies specific for the extracellular domain of EpCAM. Same cell numbers were assessed for each sample and time point, and EpCAM cell surface expression was given as mean fluorescence intensity (MFI) ratio of EpCAM versus control staining. Highest EpCAM expression values were observed in cells seeded at lowest densities (D1 and D2) and at the first day of measurement (Figure 1B, C). At higher densities, expression of EpCAM epitopes recognised by the EpEX-specific antibody decreased at the cell surface of HCT8 and MCF7 (Figure 1B, C). This decrease was even more pronounced over time for the case of HCT8 cells, which had lowest EpCAM levels at the cell surface at day 3 and under D3 conditions (Figure 1B). In HCT8 cells, an increase of EpCAM cell surface expression was seen one day post seeding under D1-2 conditions (Figure 1B). Measurement of EpCAM expression in MCF7 cells under the same conditions was restricted to a two days time span owing to the substantially bigger size of these cells as compared to HCT8 cells, which precluded further culture under those conditions. Nonetheless, significantly decreased levels of EpEX staining in MCF7 were apparent as early as day 1 (Figure 1C).

Regulated intramembrane proteolysis (RIP) is mandatory for activation of EpCAM-signalling in carcinoma cells and may represent the basis for the observed decrease of intact EpCAM molecules at the cell surface owing to EpEX shedding. Cell-free supernatants of HCT8 and MCF7 cells (400 μg each) cultured under D1, D2, and D3 conditions were immunoprecipitated with antibodies specific for the shed ectodomain EpEX. Detectable amounts of soluble EpEX were observed in the culture supernatant of HCT8 and MCF7 cells at day 1 under D2 and D3 conditions but hardly under density condition 1 (Figure 1D, E). Amounts of soluble EpEX were further increased at day 2 (Figure 1D, E). Inductions of EpEX secretion were measured upon densitometry and compared amongst cells with differing initial seeding (D2/D1 and D3/D1). Ratios were 5.8- and 20-fold for HCT8, and 7- and 23-fold for MCF7 at day 1, respectively. Inductions were 1.8- and 3.5-fold for HCT8, and 9.5- and 11-fold for MCF7 at day 2 (see n-fold ratios in Figure 1D, E). In order to further control and validate these findings, two fix cell numbers (4 × 10^5 ^and 2 × 10^6^) were each plated in culture dishes with increasing sizes resulting in starting densities comparable to D1-3 for both starting cell numbers (see Materials and Methods for details). At the time points of assessment, supernatants were equilibrated to the maximal volumes and served as a source to immunoprecipitate EpEX with specific antibodies. As for the case of differing cell numbers, EpEX shedding increased along with rising densities and resulted in similar n-fold inductions (Figure 1F and 1G). Hence, EpEX amounts shed per cell increased with enhanced densities.

**Figure 2:**
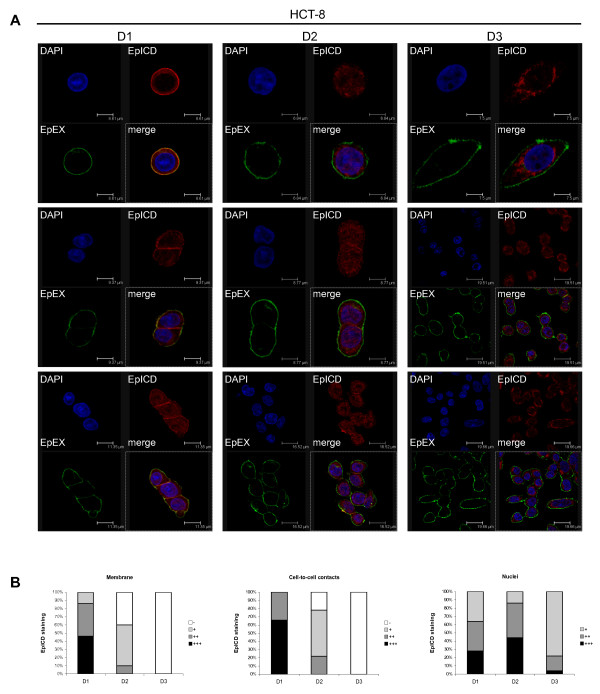
**Visualisation of EpCAM cleavage dependence on cell-to-cell contact**. (**A**) HCT8 cells were seeded at three different confluencies (D1-3), which resulted in single cells (D1), 20% (D2), and 80% confluency (D3). Thereafter, HCT8 cells were stained with antibodies specific for EpEX (green) and EpICD (red), and cellular DNA was stained with DAPI (blue). Merged pictures are displayed in the lower right quadrant of each section. Fluorescence was recorded with a laser scanning confocal microscope. Shown are three representative sections of each condition. (B) EpICD localisations were assessed semi-quantitatively and are given as percentages of cells with no (-), weak (+), intermediate (++), and strong (+++) staining at the membrane, at cell-to-cell contacts, and within the nucleus.

Next, EpCAM cleavage was monitored in carcinoma cells upon dual staining with domain-specific antibodies against EpEX and EpICD in conjunction with distinct fluorescence-labelled secondary antibodies in carcinoma cells. This allows for the visualisation of cellular re-localisation of EpICD [8]. HCT8 colon carcinoma cells at the lowest density occurred as single cells at the time point of seeding and were characterised by a co-localisation of EpEX and EpICD at the plasma membrane after one day in culture, which was indicative of intact EpCAM molecules (Figure 2A left panels). Substantial cleavage of EpCAM was seen in cells present as duplets or triplets, only. An increase of cell density resulted in cell-to-cell contact and in obvious translocation of EpICD into the cytoplasm and nucleus (Figure 2A middle and right panels). When comparing D2 and D3, nuclear translocation of EpICD appeared somewhat reduced and perinuclear accumulation was enhanced under conditions of high initial cell density (compare Figure 2A lower middle and right panels). Single cells under high-density culture conditions (D2 and D3) were nonetheless characterised by cleaved EpCAM molecules and relocalisation of EpICD within the cytoplasm and nucleus (Figure 2A, upper middle and right panels). Induction of EpCAM cleavage at the surface of single cells under these culture conditions might be explained by the presence of soluble EpEX in the supernatant of cells as early as in the D2 settings (see Figure 1D, E). Recombinant EpEX substituted for cell-to-cell contact in order to induce EpCAM cleavage in single cells while it had no effect on densely cultured cells (Additional File 1).

**Figure 3:**
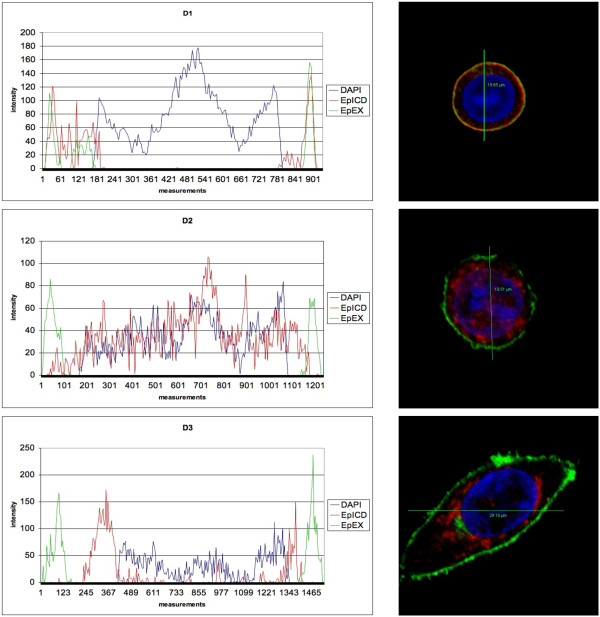
**EpEX, EpICD, and DNA subcellular localisation was quantified in HCT8 cells grown under density conditions 1-3**. Sections with a minimum of 900 measurement points were assessed per cell using the LSC Lite software. Shown are representative cells (right panel) and the according localisation plots of fluorescence intensity across cells.

EpICD sub-cellular localisation and nuclear translocation was assessed in a semi-quantitative fashion in HCT8 cells for each density. Cells cultured under D1 conditions showed a predominant membraneous localisation of EpICD, with 86% of sections (n = 50) displaying intermediate to strong EpICD staining at the plasma membrane, while only 14% of sections displayed weak staining and none of the sections showing cells entirely devoid of EpICD at the plasma membrane. Membrane staining gradually decreased with increasing cell density and resulted in cells devoid of EpICD at the plasma membrane in 100% of sections analysed under D3 (Figure 2B). This phenotype was likewise prominent when assessing EpICD localisation at cell-to-cell contacts, which was completely lost in D3 conditions (Figure 2B). Loss of membraneous localisation was paralleled by increased appearance of EpICD in the cell cytoplasm and nucleus when comparing D1 with D2 and 3. However, nuclear translocation was reduced under D3 conditions and at day 3, and displayed a strong perinuclear staining with residual nuclear staining (Figure 2, 78% weak staining in the nucleus). Similar results were obtained with the breast cancer cell line MCF7 (data not shown). Semi-quantitative data were further corroborated by a software-guided profiling of EpEX, EpICD, and DNA in confocal sections of cells cultured under differential densities. The profiles of EpEX and EpICD stainings were essentially overlaying for the case of cells culture under density D1. In contrast, EpICD localisation was cytoplasmic, perinuclear, and nuclear in cells grown under density D2 and D3 conditions. We confirmed an accumulation and enhancement of perinuclear staining, and reduced nuclear localisation of EpICD within cells kept under density D3 (Figure 3).

### Induction of EpCAM targets and proliferation

**Figure 4:**
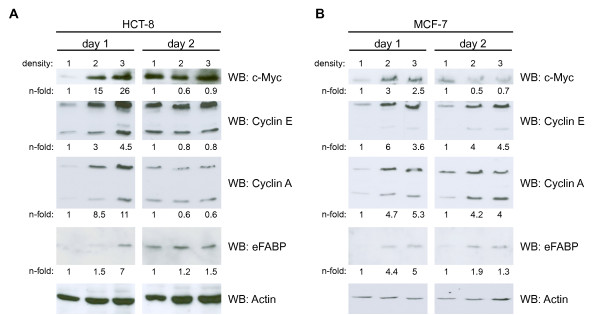
**Induction of EpCAM target genes**. HCT8 (**A**) and MCF7 (**B**) cells were seeded at three different confluencies (D1-3). At day 1 and 2 cells were lysed and the protein expression of the EpCAM target genes *c-myc*, *cyclin E *and *A*, and *efabp *was assessed upon immunoblotting with specific antibodies. For a control, actin expression levels were assessed in parallel. Levels of target genes were assessed by image densitometry, normalised for the amount of actin, and values for D1 set to one for a reference. Target gene levels are given n-fold of D1. Shown are representative results from three independent experiments.

*De novo *or over-expression of EpCAM results in the up-regulation of c-Myc, cyclins, and e-FABP, amongst others [2,22,23]. Regulated intramembrane proteolysis of EpCAM and nuclear translocation of EpCID is associated with induction of target gene expression [3,8,12]. Regulation of the target genes *c-myc*, *cyclin A *and *E*, and *e-fabp *was assessed at the protein level and in dependency of the initial cell density at the time point of seeding. c-Myc, cyclin E and A, and e-FABP expression was induced at day one in HCT8 and MCF7 cells plated at higher densities (D2 and D3) as compared to the lower density (D1, Figure 4). No substantial additional increase in target gene expression was observed at day 2 for D2 and D3, while target gene induction occurred for D1 conditions at this time point most probably owing to increased cell density (Figure 4, right panels). Levels of target gene expression in cells cultured under density D1 conditions were set to one in order to compare induction ratios across the differential densities (see n-fold numbers in Figure 4). A maximal 26-fold induction was observed for c-Myc in HCT8 cells (D3/D1) at day one.

Since cleavage and target gene induction were up-regulated in dependency of cell-to-cell contact, we analysed whether these signalling processes also resulted in changes in proliferation rates. HCT8 cells were seeded at D1, D2, and D3 and cell numbers assessed after 24 hrs and 48 hrs. In order to compare proliferation rates depending on the seeding density, doubling times (t_d_) were calculated. T_d _of cells grown under high-density conditions (D2 and D3) were significantly lower than those grown at low density (D1) at day one (D1: 0,43 ± 0,06 days; D2: 0,34 ± 0,02 days; D3: 0,34 ± 0,0 days). These differences were statistically significant: D1/D2 p = 0.04 and D1/D3 p = 0.04. At day two, cells initially seeded at D2 still had a significantly reduced t_d _as compared to D1 (D1: 0,49 ± 0,03 days; D2: 0,43 ± 0,012 days). However, cells initially seeded at D3 already reached confluency and displayed significantly increased t_d _(D3: 054 ± 0.006 days), *i.e*. a decreased proliferation rate (Table 1). These differences were statistically significant: D1/D2 p = 0.014 and D1/D3 p = 0.04. Thus, seeding density impacted on doubling times of HCT8 cells. Cells initially seeded at densities precluding cell-to-cell contact (D1) were characterised by initially higher t_d _and reciprocally by lower proliferation rates.

**Table 1:** Doubling times of HCT8 colon carcinoma cells depending on initial seeding density.

Doubling time (t_d_) in days		
**Cell number**	**t_d _24 hrs**	**t_d _48 hrs**

0.5 × 10^5^	0,43 ± 0,06	0,49 ± 0,03

3 × 10^5^	0,34 ± 0,02*	0,43 ± 0,012*

20 × 10^5^	0,34 ± 0,0*	0,54 ± 0,006*

Given are mean doubling times from three independent experiments with standard deviations.

* Significance relates to t_d _at lowest seeding density (p < 0.05).

### Cleaved EpICD is functionally independent of cell density

**Figure 5:**
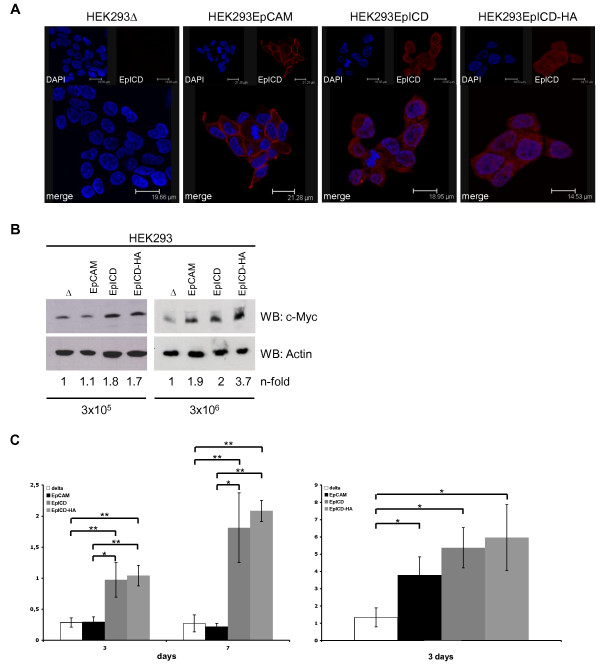
**EpICD provides target gene and proliferation induction independently of cell-to-cell contact**. (**A**) Human embryonic kidney HEK293 cells were stably transfected with empty vector, EpCAM, EpICD, or EpICD-HA expression vectors. EpICD was stained with specific antibodies (red) and nuclear DNA was stained with DAPI (blue). Shown are representative sections recorded with a laser scanning confocal microscope. (**B**) The indicated HEK293 transfectants were seeded at initial densities of 3 × 10^5 ^and 3 × 10^6 ^cells and c-Myc expression was assessed upon immunoblotting with specific antibodies. For a control, actin expression levels were assessed in parallel. Shown are representative results from three independent experiments. (**C**) The indicated HEK293 transfectants were seeded at initial densities of 3 × 10^5 ^and 3 × 10^6 ^cells. Cell numbers were assessed at day three and seven after seeding. Levels of c-Myc expression were assessed by image densitometry, normalised for the amount of actin, and values for D1 set to one for a reference. Target gene levels are given n-fold of D1. Shown are the mean cell numbers with standard deviation from three independent experiments. Significant differences are marked (* p < 0.05; ** p < 0.005).

Cell-to-cell contact was mandatory as an initial trigger to activate EpCAM *via *regulated intramembrane proteolysis (see Figure 2). Conversely, we reasoned that EpICD, when expressed as a soluble molecule, should be independent of cell density at initial seeding. In order to test this hypothesis, EpCAM-negative human embryonic kidney (HEK293) cells were stably transfected with a control vector, an EpCAM expression vector, and expression vectors encoding for EpICD and EpICD-HA. EpICD was visualised by confocal laser scanning microscopy in stable clones. Expectedly, HEK293 control cells were devoid of EpICD, while HEK293-EpCAM cells displayed EpICD staining at the plasma membrane and to lesser extent in the cytoplasm (Figure 5A). HEK293-EpICD and HEK293-EpICD-HA transfectants expressed EpICD throughout the cells, including the cytoplasm, perinuclear areas, and the nucleus (Figure 5A).

In these cell transfectants, the induction of c-Myc was monitored in dependency of the initial seeding density. A ten-fold difference in seeding density was chosen (3 × 10^5 ^and 3 × 10^6 ^in a 10 cm culture dish format). At low seeding density, EpCAM expression did not influence c-Myc expression as compared with control cells (ratio EpCAM/control = 1.1), while EpICD and EpICD-HA expression associated with a two-fold increase in c-Myc levels (Figure 5B left panel). In contrast, EpCAM expression induced enhanced levels of c-Myc when cells were seeded at higher density, which allowed for cell-to-cell contact. Under these conditions, induction of c-Myc by EpCAM, EpICD, and EpICD-HA was similar and superior to EpCAM-negative control cells (Figure 5B right panel; 1.9-, 2-, and 3.7-fold, respectively).

Next, we compared the effect of EpCAM and EpICD on cell proliferation depending on the initial cell density in the genetic background of EpCAM-negative HEK293 cells. As for the induction of c-Myc, EpCAM expression resulted in increased cell numbers as compared to control transfectants only under high-density conditions (Figure 5C, right panel). EpICD and EpICD-HA remained unaffected by cell densities in their capacity to increase cell proliferation. EpICD-positive cells steadily grew to numbers three-fold higher than control cells at day three, independently of the seeding density (Figure 5C, left panel). Hence, EpICD was independent of cell-to-cell contact with respect to its capacity to induce a proliferation phenotype in HEK293 cells, while EpCAM requires sufficient initial density.

### Nuclear translocation is mandatory to develop EpICD effects

**Figure 6:**
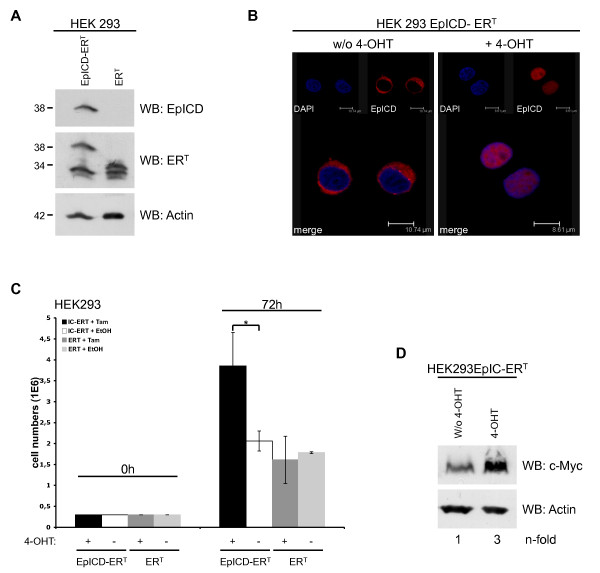
**EpICD nuclear translocation is mandatory for function**. (**A**) HEK293 cells were stably transfected with ER^T ^or EpICD-ER^T ^expression plasmids. Expression of ERT and EpICD-ERT was assessed upon immunoblotting with ER- and EpICD-specific antibodies in combination with HRP-conjugated secondary antibody. Shown are representative results from three independent experiments. (**B**) HEK293 EpICD-ERT were treated with 4-hydroxytamoxifen (4-OHT, 100 nM) or diluent only (w/o 4-OHT) for 1 hr. EpICD was stained with specific antibodies (red) and nuclear DNA was visualised with DAPI (blue). Sections were recorded with a laser scanning confocal microscope. Shown are representative images from three independent experiments with multiple sections each. (**C**) HEK293ER^T ^and HEK293EpICD-ER^T ^cells were plated (3 × 10^5^/plate) and cultured in the presence or absence of 4-OHT for three days. Cell numbers were assessed upon trypan blue counting and mean numbers with standard deviations from two independent experiments are given (* p < 0.05). (**D**) HEK293EpICD-ER^T ^cells were treated with 4-hydroxytamoxifen (4-OHT, 100 nM) or diluent only (w/o 4-OHT) and c-Myc expression was assessed upon immunoblotting with specific antibodies and HRP-conjugated secondary antibodies. For a control, actin expression levels were assessed in parallel. n-fold induction of c-Myc was assessed upon densitometry and is given as the ratio of tamoxifen- versus control-treated cells. Shown are representative results from two independent experiments.

Although suggested by data available so far, an experimental proof of a requirement of nuclear translocation of EpICD in order to deploy oncogenic effects is still lacking. We established a cellular system to test this notion *in vitro*. EpICD was fused to a mutated ligand-binding domain of the human estrogen receptor, which reacts to tamoxifen, in order to generate EpICD-ER^T^. As a control the ER^T ^moiety was transfected in HEK293 cells. Both proteins were visualised upon immunoblotting with EpICD- or ER-specific antibodies (Figure 6A). In the absence of the estrogen analog 4-hydroxytamoxifen (4-OHT), the EpICD-ER^T ^fusion is retained in the cytoplasm. Upon addition of 4-OHT to culture media, EpICD-ER^T ^is relieved from binding to chaperones and can translocate into the nucleus (Figure 6B). Control ER^T ^and EpICD-ER^T ^transfectants were compared for their proliferation capacity by cell counting. After four days, control ER^T ^grown in the presence or absence of 4-OHT and EpICD-ER^T ^cells grown in the absence of 4-OHT did not differ in cell numbers (Figure 6C). Induction of EpICD-ER^T ^nuclear translocation however resulted in a mean increase of cell numbers by a factor of two (Figure 6C), and was paralleled by 3-fold enhanced c-Myc protein levels (Figure 6D). These effects and values were similar to phenotypes observed upon de novo expression of full length EpCAM in HEK293 cells [2].

## Discussion

More and more, a dual role for EpCAM becomes evident as was long known for cadherins and proteins of the immunoglobulin superfamily [24,25]. EpCAM is a transmembrane protein engaged in cell adhesion [26] and nuclear signalling [8,9], which is instrumental in cell proliferation and morphoregulation [27,28]. Generally speaking, high expression of EpCAM associated with a proliferative and regenerative phenotype in normal tissues [29-31] with active sites of cell division, and with cancer-initiating cells in tumours *in vivo *[32,33]. Eventually, EpCAM is reckoned as a potent oncogenic factor, which is activated via regulated intramembrane proteolysis [8,9] and which plays an important role in cancer and stem cell signalling [10-12]. In this respect, the remarkably short intracellular domain termed EpICD is necessary and sufficient to deploy oncogenic effects *in vitro *and in animal models of cancer [2,8].

In the present work, we have assessed the actual need for cell-to-cell contact for the activation of EpCAM cleavage *via *regulated intramembrane proteolysis. Upon variation of cell densities, it became clear that cell-to-cell contact was involved in the initial activation of EpCAM signal transduction by cleavage. If cellular contact was allowed, then cleavage proceeded, was sufficient to generate soluble EpEX, to release EpICD from its membrane-associated localisation, and to induce target genes. Soluble EpEX conferred cleavage to single cells in the culture under these conditions. This type of initial juxtacrine activation following cell-to-cell contact may allow for a restricted radius of strong effects and may achieve fine patterns of cellular cross-talk [16]. The ability to create a soluble ligand in the form of shed EpEX provides even more flexibility. Upon initial local contact, cells generate a means for long range paracrine signalling including a gradient of activation. Interestingly, EpCAM interacts with CD44 [34], itself a transmembrane protein that becomes cleaved, which hence appears as a common theme of receptor co-activation that is apparently governed by tetraspanins and associated proteins [35-37]. The assembly and disruption of such complexes is yet another level of regulation of signalling, which *in vivo *might be affected by cell-to-cell contacts. Certainly, the differential localisation of EpCAM in normal tissue (basolateral) versus carcinomas (homogenous distribution at the membrane) will further influence EpCAM interactions and activation.

Cleavage and nuclear translocation of EpCAM is associated with an induction of target genes [2,23] and with reduced doubling times given the fact that cells were not contact inhibited. Thus, EpCAM interactions may allow a sensing of the presence of cognate cells to trigger proliferation *via *EpCAM cleavage and nuclear translocation of EpICD such as in condition D2. This would rather mimic the state of micrometastasis and of normal stem/progenitor cells, *e.g*. when repopulating injured organs. Notably, the regeneration of damaged liver and kidney were conducted by progenitor cells, which re-expressed EpCAM to high levels. Upon differentiation to hepatocytes and completion of organ regeneration, EpCAM expression is lost again [31,38,39]. In case neighbouring cells are missing, like it is *e.g*. the case for disseminated tumour cells, cells might decrease levels of EpCAM activation, receive less proliferation signals, and might rest in a state of quiescence (condition D1). For the case of contact inhibition of cell proliferation, although cleavage of EpCAM occurred to high extent, nuclear translocation was reduced and EpICD accumulated as peri-nuclear speckles besides nuclear speckles. Nonetheless, these remaining levels of nuclear EpICD were instrumental for the rapid induction of target genes, which reached a plateau at day two of assessment. For the case of MCF-7 cells, which are bigger than HCT-8, a reduction of the target gene c-Myc was observed under D3 conditions already at day 2 and might hence be anticipated for HCT-8 at later time points. These imaging results were paralleled by corroborative cells numbers. Cells grown under high density conditions only displayed low doubling-times at day one, with a sharp increase of doubling times at day two. Inhibitory effects impacting on EpICD nuclear translocation might further account for differences in localisation as observed *in vivo *in normal colonic mucosa and colon carcinomas [8].

The molecular basis for this partial reduction of EpICD nuclear translocation and its potential implication in the regulation of EpCAM effects are totally unclear and deserve further research. Most probably, effects of EpCAM on proliferation are overcome and dampened by mechanisms of contact inhibition, which might even actively impair on EpICD nuclear translocation, *e.g. via *cytoplasmic inhibitors which retain EpICD. This means of regulation of EpCAM effects at the level of subcellular localisation was further underscored by experiments using conditional systems *in vitro*. For the first time, targeted translocation of EpICD demonstrated the necessity of nuclear localisation in order to deploy regulatory effects on the expression of c-Myc and on cell proliferation.

In summary, EpCAM becomes cleaved upon cell-to-cell contact in a juxtacrine manner and additionally fosters signalling in a paracrine fashion using soluble EpEX.

## Conclusion

Activation of EpCAM's cleavage and oncogenic capacity is dependent on cellular interaction (juxtacrine) to provide for initial signals of regulated intramembrane proteolysis, which then support signalling via soluble EpEX (paracrine).

## Abbreviations

EpCAM: epithelial cell adhesion molecule; EpEX: EpCAM extreacellular domain; EpICD: EpCAM intracellular domain; 4-OHT: 4-hydroxy tamoxifen; ER: estrogen receptor; HA: haemagglutinin tag; D: density

## Competing interests

The authors declare that they have no competing interests.

## Authors' contributions

SD carried out cell and molecular biology studies; BM carried out cell imaging experiments; DM carried out cell and molecular biology studies, and participated in the coordination of the study; CE and GB participated in cell and molecular biology experiments; OG conceived the study, participated in its design and coordination, and wrote the manuscript.

## Pre-publication history

The pre-publication history for this paper can be accessed here:

http://www.biomedcentral.com/1471-2407/9/402/prepub

## Supplementary Material

Additional file 1 **Localisation of EpEX and EpICD following treatment of EpCAM-positive carcinoma cells with recombinant EpEX**. Where indicated, HCT-8 were treated with 1 μg rEpEX before staining with EpEX- and EpICD-specific antibodies (green and red, respectively). DNA was stained with DAPI (blue). Sections were recorded with a laser scanning confocal microscope. Shown are representative images from two independent experiments with multiple sections each. Click here for file
