# Supplementary figures and images for: Initial activation of EpCAM cleavage via cell-to-cell contact
Source: BMC Cancer. 2009 Nov 19;9:402. doi: 10.1186/1471-2407-9-402 (PMC2784796; doi:10.1186/1471-2407-9-402)

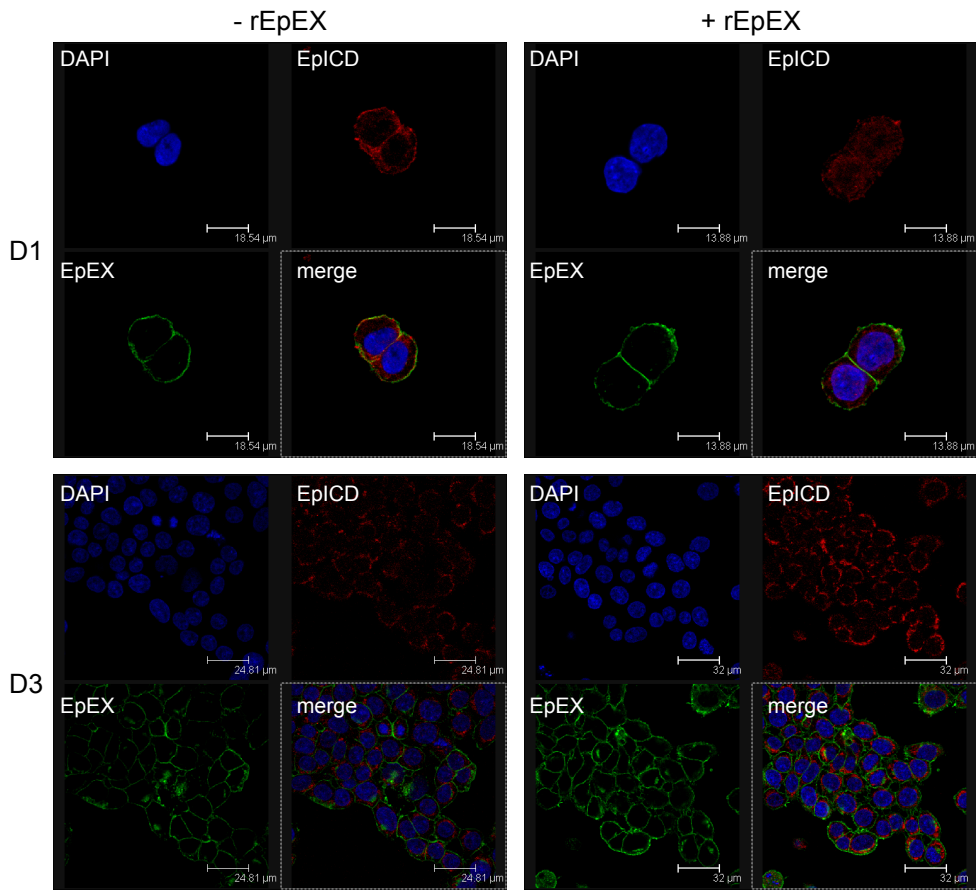

Supplement: Additional file 1 — Localisation of EpEX and EpICD following treatment of EpCAM-positive carcinoma cells with recombinant EpEX. Where indicated, HCT-8 were treated with 1 μg rEpEX before staining with EpEX- and EpICD-specific antibodies (green and red, respectively). DNA was stained with DAPI (blue). Sections were recorded with a laser scanning confocal microscope. Shown are representative images from two independent experiments with multiple sections each. [file 1471-2407-9-402-S1.PDF]
